# Stunted root development: A rare dental complication of Stevens-Johnson syndrome

**DOI:** 10.4317/jced.52826

**Published:** 2016-10-01

**Authors:** Aditi Sangwan, Hans-Raj Saini, Pankaj Sangwan, Parveen Dahiya

**Affiliations:** 1 Senior Resident, Dept. Of Periodontics, PGIDS, Rohtak; 2 Senior Demonstrator, Dept. Of Conservative Dentistry, PGIDS, Rohtak; 3 Professor, Dept. Of Conservative Dentistry, PGIDS, Rohtak; 4 Reader, Dept. of Periodontics, Himachal Institute of Dental Sciences, Paonta Sahib

## Abstract

Stevens-Johnson syndrome (SJS) is a severe cutaneous reaction seen rarely in clinical practice. Most often, it occurs as an adverse reaction to certain drugs. When it affects children at a very young age, arrested tooth root development may also be seen. We present a case of a 13 year old boy who suffered from SJ syndrome at the age of 7 years. Incomplete root development was observed in all teeth, as demonstrated by panaromic radiography. Clinical features of this condition and its management are further discussed. We aim to emphasise on the need for dental practitioners to be aware of the potential dental complications of SJS and enable them to recognise and manage the condition at the earliest so as to avoid any undesirable sequelae.

** Key words:**Adverse drug reaction, amoxycillin, arrested root development, Stevens-Johnson syndrome.

## Introduction

Antibiotics, while often life saving, have the potential to cause certain adverse effects which might, at times, be serious in nature. Stevens-Johnson syndrome (SJS) is one such acute reaction which has been associated with morbidity and mortality. However, it is rare with an extremely low incidence of about 2 cases per 1 million populations per year (1). While numerous articles have been published emphasising the systemic and general physical consequences of SJS, there is a dearth of literature dealing specifically with the dentofacial outcomes of such a reaction. Abnormal root development, first reported in 1979 by De Man (2), is yet another infrequently reported complication. Amoxicillin is a frequently used drug and is relatively well tolerated by patients. The case presented here pertains to the dental complications of SJS observed in a child following the administration of amoxicillin. The purpose of this case report is to provide an insight into the features and management of SJS and spread awareness regarding its potential implications in the dental field.

## Case Report

A 13 year old boy reported to the Department of Conservative Dentistry, Post Graduate Institute of Dental Sciences (PGIDS), Rohtak with the complaint of sensitivity in his teeth. His parents wanted to him to undergo dental treatment before taking him abroad for corneal transplant (also a sequela of SJS). On obtaining detailed history, it was revealed that the boy had suffered from an acute adverse drug reaction after taking a course of amoxycillin, which was advised by a physician when the child was seven years old. He developed fever with generalised erythema and desquamation of skin along with painful ulcerations in the mouth and eyes. He was then referred to another physician who diagnosed the condition as SJS. The patient was hospitalised for around one month and was administered hydrocortisone among other drugs to control the reaction. According to the child’s parents, while the skin lesions healed and other problems resolved, vision could not be restored. They were now planning for a corneal transplant, which had been suggested for restoration of the child’s vision.

**Figure 1:**
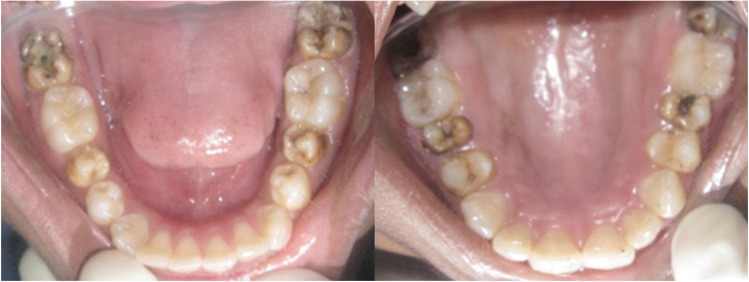
Intraoral photograph showing clinical status of teeth.

**Figure 2:**
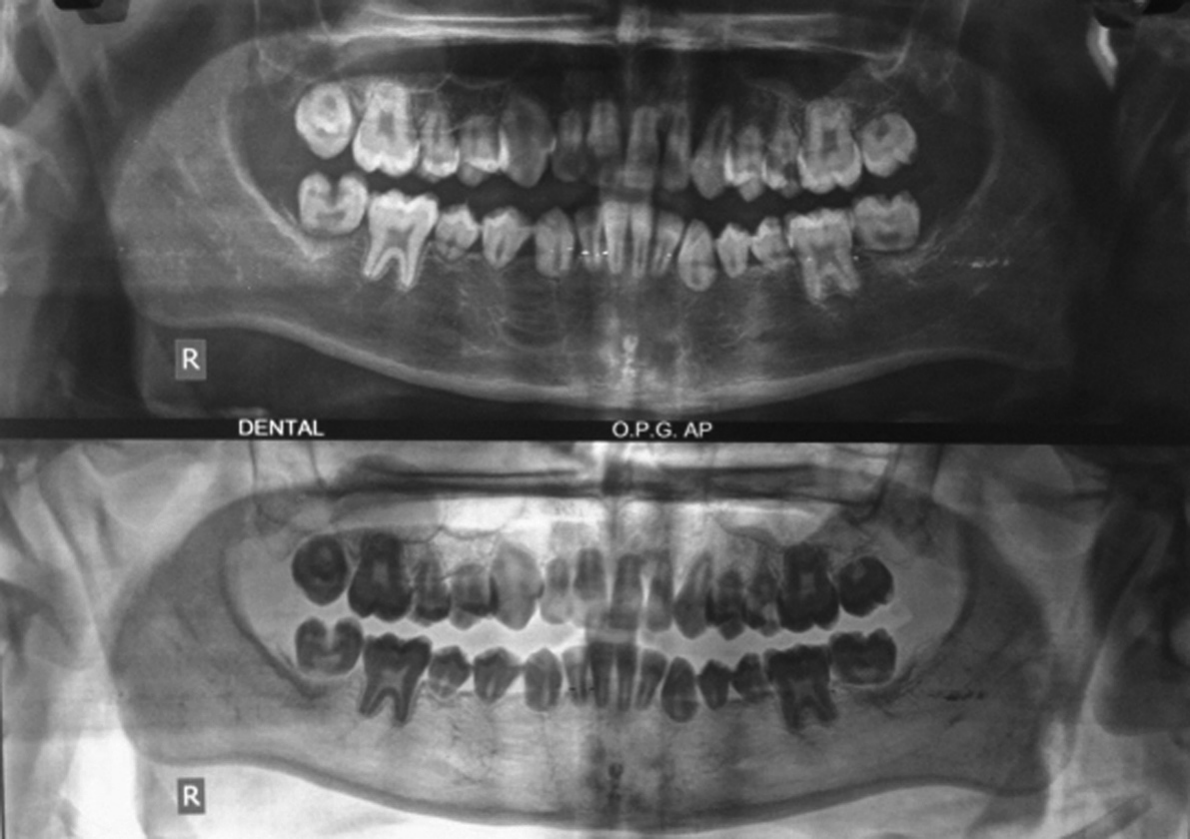
OPG showing incomplete root development of almost all the teeth.

On intraoral examination, the patient had all the teeth erupted up to second molars in both the arches (Fig. 1). Five of his teeth viz. 15, 25, 37, 47 and 45 were carious. Caries in 37 and 47 were deep, for which IOPA radiographs were advised. Both the radiographic images revealed incomplete root development of the involved as well as adjacent teeth. A panaromic radiograph was then advised, which displayed incomplete root development of almost all the teeth (Fig. 2). The premolars and second molars were the worst affected, with almost negligible root formation. Even though the root development was not complete, all teeth responded positively to thermal and electric pulp tests. Since there was no history of irreversible pulpitis, the teeth were permanently restored.

Written informed consent for publication was provided by the patient’s parent and ethical approval taken from Institutional Ethics Committee of PGIDS, Rohtak.

## Discussion

Steven and Johnson first described the eponymous syndrome in 1922 in two cases presenting with fever, severe generalised eruptions on skin, stomatitis and opthalmia (3). It is considered to be a variant of Toxic Epidermal Necrolysis (TEN), with the two conditions differentiated primarily on the basis of severity and body surface area involved. An immune reaction with epithelial involvement of less than 10% of body surface area (BSA) is classified as SJS (4). SJS often begins with nonspecific influenza- like symptoms such as fever, malaise, myalgias and arthralgias that may last anywhere from 1 to 14 days. The skin lesions, which appear suddenly and symmetrically on the body, are macular, ill defined and tender and may coalesce with the passage of time. The epidermis in affected areas undergoes necrosis and becomes loose and easily detachable, especially at sites of pressure or friction. Involvement of oral mucosa is seen in most of the cases with widespread and painful ulcerations. Burning sensation, edema and erythema of the lips and buccal mucosa may be evident before the actual appearance of mucosal erosions. While buccal and palatal mucosa along with lips are the most commonly affected areas in the oral cavity, involvement of entire oral cavity, pharynx, and even esophagus or trachea may also be seen. Clinically, the lesions present as severely painful ulcerated mucosa covered by necrotic debris and hemorrhagic crusts (5).

The most indispensable part of the management of SJS involves immediate withdrawal of all drugs which may be suspected to have caused the reaction. Since the reaction is immune in nature, the pharmacological management must be aimed at reducing the severity of reaction and decreasing the extent of involvement of skin and internal organs. Although steroids are still considered to be the backbone of pharmacological management of SJS, there are many who firmly stand opposed to the use of steroids in this condition (5). Of late, intravenous administration of immunoglobulins has been suggested as an alternative to the now less preferred corticosteroids (6). Equally important is supportive therapy in the form of fluid and electrolyte replacement, adequate nutrition and care of affected muco-cutaneous areas.

While generalised shortening of tooth roots is rarely encountered in clinical practice, it has previously been reported in metabolic disorders associated with deranged calcium metabolism such as hypoparathyroidism, haematological conditions like thalassemia (indirectly resulting in calcium deficiency due to endocrine insufficiency) and even neurological disorders like epilepsy (in cases on long-term phenytoin therapy leading to disturbance of calcium metabolism ) (7-9). A condition called short root anomaly, also called as root dwarfism or rhizomicry which most commonly affects the maxillary central incisors is ascribed to genetic hypodontia (10). Two other hereditary autosomal dominant conditions which are often associated with shortened roots include dentinogenesis imperfecta and radicular dental dysplasia (11). Localized root shortening due to inflammation has also been attributed to trauma, local periapical inflammation, periodontal diseases as well as a side-effect of orthodontic treatment (12). All of the above mentioned conditions must be considered in the differential diagnosis of the present case. Several factors helped in differentiating the present case from the above mentioned conditions. No positive family history or other relevant signs and symptoms were recorded in the present case. Serum calcium and parathormone levels were found to be normal. Moreover, one peculiarity specifically set apart the present case from the other conditions. The roots in this case were not uniformly short; the roots of incisors and first molars were nearer to completion as compared to the other teeth. The whole picture gave an impression that the root formation had been proceeding normally till the time some unfavourable event abruptly led to complete cessation of the process. In consideration of the developmental stages of roots of different teeth, it appears that root cessation occurred at around the age of 7 to 8 years. This coincides with the age at which the patient suffered from SJS. The only reasonable explanation for this arrested root development is that the Hertwig’s epithelial root sheath, which is the epithelial proliferation zone in developing teeth, must have got damaged during the immune reaction. This argument corroborates with the findings of Gautlier *et al.* (13) who conducted an oral and dental examination of sixteen patients with SJS/TEN. They reported similar root abnormalities in three of their patients and suggested that in such a reaction, the Hertwig’s epithelial root sheath cells get destroyed by an acute process of apoptosis and result in root abnormalities.

It was, however, surprising that despite the root abnormalities, the teeth showed no signs of mobility and all reached almost to the occlusal level. Since the teeth were functional and there was no sign of pulpal involvement, a decision was taken to restore the teeth.

## References

[1] Rzany B, Mockenhaupt M, Baur S, Schroder W, Stocker U, Mueller J (1996). Epidemiology of erythema exsudativum multiforme majus, Stevens-Johnson syndrome, and toxic epidermal necrolysis in Germany (1990-1992): structure and results of a population-based registry. J Clin Epidemiol.

[2] De Man K (1979). Abnormal root development probably due to erythema multiforme (Stevens-Johnson syndrome). Int J Oral Surg.

[3] Anna WM, Pierce WW (1949). Stevens-johnson syndrome; eruptive fever with stomatitis and ophthalmia. N Y State J Med.

[4] Bastuji-Garin S, Rzany B, Stern RS, Shear NH, Naldi L, Roujeau JC (1993). Clinical classification of cases of toxic epidermal necrolysis, Stevens-Johnson syndrome, and erythema multiforme. Arch Dermatol.

[5] Fritsch PO, Sidroff A (2000). Drug-induced Stevens-Johnson syndrome/toxic epidermal necrolysis. Am J Clin Dermatol.

[6] Jolles S, Sewell WA, Misbah SA (2005). Clinical uses of intravenous immunoglobulin. Clin Exp Immunol.

[7] Kamarthi N, Venkatraman S, Patil PB (2013). Dental findings in the diagnosis of idiopathic hypoparathyroidism. Ann Saudi Med.

[8] Hazza'a AM, Al Jamal G (2006). Radiographic features of the jaws and teeth in thalassaemia major. Dentomaxillofac Radiol.

[9] Jindal G, Pandey RK, Kumar D (2012). Generalised stunting of roots in an epileptic child: is long-term phenytoin therapy the cause?. BMJ Case Rep.

[10] Apajalahti S, Hoitta P, Turtola L, Pirinen S (2002). Prevalence of short-root anomaly in healthy young adults. Acta Odontol Scand.

[11] Barron MJ, McDonnell ST, MacKie I, Dixon MJ (2008). Hereditary dentine disorders: dentinogenesis imperfecta and dentine dysplasia. Orphanet Journal of Rare Diseases.

[12] Brezniak N (2002). Orthodontically inducted inflammatory root resorption. Part II: The clinical aspects. Angle Orthod.

[13] Gaultier F, Rochefort J, Landru MM, Allanore L, Naveau A, Roujeau JC (2009). Severe and unrecognized dental abnormalities after drug induced epidermal necrolysis. Arch Dermatol.

